# Observations Respecting the Sensible Perspiration of the Dictamnus Albus, or Fraxinella

**Published:** 1811-06

**Authors:** Robert Lyall

**Affiliations:** Surgeon, M.R.P.S.E. &c.


### *Observations respecting the Sensible Perspiration of the Dictamnus Albus, or Fraxinella. By Mr.* Robert Lyall, *Surgeon, M.R.P.S.E. &c.*

(Nichol. Jour.)

It has been said, that in calm summer evenings the dictamnus albus evolves hydrogen gas, or a highly odorous inflammable effluvium, which explodes when brought into contact with the flame of a candle; an opinion that is maintained in the latest botanical publications I have seen.

When I first became acquainted with the above notion, my curiosity was excited, and I longed for an opportunity to make the experiment, which was not very long denied me. The result of my observations I shall now relate in order, that the subject may be more accurately investigated.

I need scarcely premise, that the peduncles, the calyx, the outside of the corolla, and especially the tops of the filaments, and the germen of the dictamnus, are covered with glands of an oblong form, many of them supported on little pedicles, all of them of a beautiful red colour, and containing a somewhat viscid fluid.

On the 10th of July, about ten in the evening, the weather fine, and the temperature 66, I commenced my experiments on the dictamnus. By holding a lighted candle at the bottom of a raceme of flowers, inconsiderable explosions, or rather a hissing noise was occasioned, accompanied by light-blue coloured flame, which proceeded along the course of the peduncles, peduncles, &c. and ascended even higher than the top of the stem; a good deal resembling an amusing experiment sometimes practised in the theatre, and often by boys, by means of powdered resin and a burning candle, &c. Immediately after the combustion, the surrounding atmosphere became tainted with odoriferous effluvia, exactly similar to what the healthy flowers, though much stronger, emit. July 13, I repeated this experiment, at the same hour as before. The evening was fine, but the plants were wet with the afternoon's rain. Scarcely any noise was produced; the experiment not succeeding as before.

At another time I brought home a raceme of flowers, and after it had stood with its end placed in water for two hours, I approached a burning candle to it, and little explosions followed. I replaced the raceme in the water, and next morning darkened my room, and made the same experiment, but heard no explosions. Since the 13th of July, I have frequently repeated the first experiment, but never have succeeded nearly so well as at first; a little hissing noise, attended with a small flame, only occurring now and then, occasioned in consequence of the bursting, I imagine, of the glands of new flowers; which, from their not being before developed, remained uninjured, during former experiments.

On examining the plants after the combustion, I observed that the glands were completely destroyed; and thus I was led to suppose, that the resinous fluid which they contained was burnt during the explosion; and not that hydrogen, or any inflammable vapour was exhaled. Since after-experiments never succeeded so well as the first; and because the smell of hydrogen was never present, either before or after the experiment, I think I am strengthened in my opinion. At the same time, however, I confess, that I am not completely satisfied with my own observations, and therefore wish that some one, who has convenience, would not only repeat the experiments, but communicate the result of them to the public, and thus either ascertain the truth of what I have reported, or annul it altogether.

